# SWI/SNF complex, promising target in melanoma therapy: Snapshot view

**DOI:** 10.3389/fmed.2023.1096615

**Published:** 2023-02-09

**Authors:** Mahsa Mollapour Sisakht, Mohammad Amir Amirkhani, Mohammad Ali Nilforoushzadeh

**Affiliations:** ^1^Biotechnology Research Center, Faculty of Pharmacy, Tehran University of Medical Sciences, Tehran, Iran; ^2^Department of Biochemistry, Erasmus University Medical Center, Rotterdam, Netherlands; ^3^Skin and Stem Cell Research Center, Tehran University of Medical Sciences, Tehran, Iran

**Keywords:** melanoma, SWI/SNF enzymes, epigenetics, chromatin remodeling, synthetic lethality, cancer therapy

## Abstract

Therapeutic strategies based on epigenetic regulators are rapidly increasing in light of recent advances in discovering the role of epigenetic factors in response and sensitivity to therapy. Although loss-of-function mutations in genes encoding the SWItch/Sucrose NonFermentable (SWI/SNF) subunits play an important role in the occurrence of ~34% of melanomas, the potential of using inhibitors and synthetic lethality interactions between key subunits of the complex that play an important role in melanoma progression must be considered. Here, we discuss the importance of the clinical application of SWI/SNF subunits as a promising potential therapeutic in melanoma.

## 1. Introduction

The SWI/SNF complex is a large and evolutionarily conserved chromatin remodeler whose epigenomic changes are characterized in most cancers. This complex consists of 15 subunits encoded by 28 genes, including *SMARCB1* (also known as *SNF5, BAF47*, and *INI1*), *SMARCC1/SMARCC2* (also known as BAF155 and BAF170), and one of the two mutually exclusive ATPase subunits, *SMARCA4* (also known as BRG1) and *SMARCA2* (also known as BRM), which are commonly mutated in 20% of human cancers (1). Although several studies show the function of this complex as a transcriptional regulator, both tumor suppressive and enhancing functions of this complex have been investigated depending on the context. In some patients with hepatocellular carcinoma, ARID1A was strongly expressed in primary tumors but not in metastatic lesions, suggesting that ARID1A may be lost after initiation. Mechanistically, enhancement of ARID1A function promoted initiation by increasing cytochrome P450-mediated oxidative stress, while loss of Arid1a in tumors decreased chromatin accessibility and reduced transcription of genes associated with migration, invasion, and metastasis. At the same time, metastasis was reduced *via* transcriptional regulation of *EMILIN1/MAT1A/LCN2/IL1R1 in vitro*. Conversely, loss of ARID1A may increase the risk of steatohepatitis and cancer progression by altering immunity *in vivo* or tumorigenesis *via* activation of angiopoietin-2 (ANGPT2) transcription *in vitro* and angiogenesis *in vivo* (2). In summary, ARID1A, as a component of the SWI/SNF complex, plays a context-dependent tumor suppressive and oncogenic role in cancer (3). Melanoma results from the malignant transformation of certain cells called melanocytes. These cells are derived from multipotent cells of the neural crest and are responsible for melanin production (4). Metastatic melanoma is a highly aggressive malignancy that responds poorly to chemotherapeutic agents. Although targeted therapy with immune checkpoint inhibitors has resulted in significant improvement in tumor control, many patients do not respond to therapy, making it necessary to identify new therapeutic targets for patients (5). Despite the improvement in therapies developed for melanoma, the 10-year survival rate for patients with advanced melanoma is ~10% (6). The SWI/SNF component has been shown to play a critical role that can be targeted to develop a new therapeutic strategy (7). The synthetic lethal effect of the SWI/SNF subunits, demonstrated in several studies, has opened the possibility for new therapies. In light of the previous study, we attempt in this review to simplify and focus on the major subunits of the SWI/SNF complex in melanomagenesis that influence sensitivity to therapeutic agents. This review summarizes recent publications to highlight the most important SWI/SNF components based on statistical analyses related to melanoma progression and resistance and/or response to current therapies associated with this complex.

## 2. SWI/SNF complex: Structure and function

**Figure 1:**
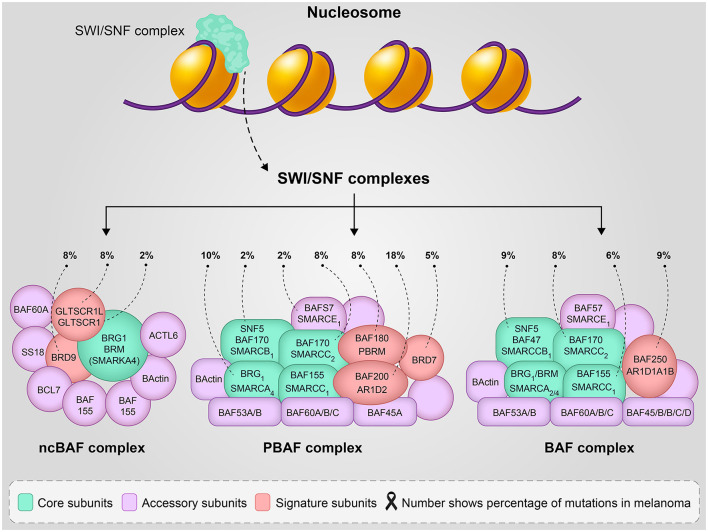
The SWI/SNF complexes structure and function, number shows the percentage of mutation involvement in melanoma reported in reference (5).

SWI/SNF is the first identified ATP-dependent chromatin remodeling multicomponent complex (consisting of 4–17 subunits) (8) that regulates the expression of 5% of genes in yeast (9) and plays an important role in transcription, DNA replication, and repair. This complex has a central catalytic subunit which is SMARCA4 (BRG1) or SMARCA2 (BRM) in the BAF complex, and 10–13 associated subunits (4), SMARCA4 or SMARCA2 function as catalytic subunits of other complexes called canonical (c) BAF, polybromo-associated BAF (PBAF) or non-canonical (nc)BAF (Figure 1). The different biological activity of these complexes is not fully understood, several functions of the biological activity of the complex are described by the genetic deletion of its subunits. The polybromo-associated BAF complex (PBAF) can be distinguished from the cBAF (canonical BAF complex) by the inclusion of BAF200 instead of BAF250A/B and BAF180 (10).

The components of the BAF complex can have cell type-specific functions, as evidenced by KD (knock-down) or KO (knock-out) of various subunits that had lethal effects, especially during embryogenesis (11, 12). The function of the BAF complex (esBAF) in pluripotency of embryonic stem cells was demonstrated by HO et al. in 2009 (13), and in 2019, the role of the non-canonical BRD9-containing BAF complex in regulating pluripotency in mouse embryonic stem cells was published (14). In addition, the function of other parts of the BAF complex, such as Baf60c, in the reversion of BAF to heart-specific enhancers during organogenesis (15) and in the differentiation of serotonergic neurons (16) has been published. All SWI/SNF components involve protein-protein or DNA-binding domains that are important for chromatin targeting and remodeling. Based on the biological activity of the complex, this complex contains three functional parts: the actin-related protein (ARP) module as a regulator, Snf2 (ATP-dependent motor that drives the basic DNA translocation reaction), and the substrate recruitment module, SRM. Recently, He and colleagues demonstrated the 12-member SWI/SNF complex *in vitro* by overexpressing the individual subunits in E. coli using the modified EcoExpress system to show the functional modularity of these complexes (9). Recent genomic studies have shown that the BAF and PBAF complexes are the most frequently mutated in various cancers (17). Non-canonical complexes (ncBAF) localize to the transcriptional repressor CTCF (also known as 11-zinc finger protein or CCCTC-binding factor) and can act as synthetic lethal targets specific for synovial sarcoma (SS) and malignant rhabdoid tumor (MRT) (16) or other cBAF-disrupted cancers.

## 3. SWI/SNF component in melanoma

Loss-of-function mutations in the components of the SWI/SNF complex such as AT-rich interactive domain-containing protein 1A (ARID1A), ARID1B, ARID2, or SMARCA4 are common in melanoma, suggesting that altered chromatin remodeling plays a role in the pathogenesis of this disease (18). A recent comprehensive study published by Dreier et al. using data from the TCGA showed a comparison of the frequency of genetic SWI/SNF alterations in melanoma. Interestingly, the data showed that the ARID2 subunit with missenses and truncating mutations is the most frequently mutated subunit in melanoma and is associated with a UVB mutation signature. Then, ARID1B missense mutations and deep deletions were observed in skin and choroidal melanomas, respectively. SMARCA4 and its paralogue SMARCA2 are almost equally frequently mutated (mostly as missenses) in patients with melanoma (5, 19). Missense mutations in these two tumor suppressors are associated with damage from UV radiation, as the role of SMARCAs is to promote melanin synthesis to protect against damage from solar radiation (20). Based on this study, ARID1A is the third most frequently mutated SWI/SNF, present in 9% of melanoma tumors in the TCGA database and associated with late-stage melanoma and metastasis to the brain (5) (Figure 1). On the other hand, several studies suggest that overexpression of the wild-type of ARID1A in patients with melanoma is more responsive to immune checkpoint inhibitors, while patients with loss of ARID1A may have therapeutic implications by modulating response to immunotherapy (21–23). However, a recent cohort study showed that ARID1A mutations do not have a major impact on survival and particularly on immune checkpoint inhibitors in melanoma. This finding by Griewank et al. (24). showed that ARID1A mutation status in melanoma is not currently relevant to treatment. Recent evidence suggests that ARID2, as a component of the PBAF complex, functions to inhibit invasion *in vitro* and modulate the response to immunotherapy *in vivo* (25). The mechanism of ARID2 as a modulator of tumor immunity is still unclear. The 2021 study by Fukumoto et al. showed that ARID2 knockout sensitizes melanoma to immune checkpoint inhibitors (anti-PD-L1 treatment), which is associated with an increase in the infiltration of CD8^+^ cytotoxic T cells. These results indicate that ARID2 is an immunomodulator and a potential biomarker indicating the efficacy of immune checkpoint inhibitors in patients with melanoma (25). In addition, mutations (C > T-transitions) in ARID2, PPP6C, SNX31, and TACC1 possibly played a role in UVB-induced melanomagenesis (18).

### 3.1. Synthetic lethal partners

Synthetic lethality is a concept used as one of the most interesting, effective, and safe strategies in cancer treatment. It aims to target alleles of genes with loss-of-function mutations by drug inhibition, deletion, or reduction of expression to induce cell death. One of the best-known agents targeting inhibition of specific DNA repair pathways, based on the synthetic lethal approach, is the use of poly(ADP-ribose) polymerase (PARP) inhibitors to target BRCA1/2 mutated tumors (26). A 2019 systematic review by Kubicek et al. demonstrated strong synthetic-lethal interactions between two factors, SMARCA4-ARID2, in subunits of the SWI/SNF chromatin remodeling complex in melanoma. In view of this theory, cancer cells that have a LOF gene mutation lead to cell death when two genes are lost simultaneously, but not when either gene is lost alone, as we have described above. Thus, the relationship between SMARCA4 and ARID2 would be a potential target for synthetic lethal therapy, as SMARCA4 and ARID2 are frequently mutated in melanoma (27). On the other hand, in cancer cells lacking SMARCA4, SMARCB1, ARID1A, and PBRM1 of the SWI/SNF chromatin remodeling complex, inhibition of EZH2 (subunits of Zeste 2 Polycomb Repressive Complex 2; such as tazemetostat^1^ or valemetostat) can cause synthetic lethality (19).

ARID1B, as a component of a subset of cBAF complexes, is a homolog of ARID1A that promotes a compensatory pathway in the event of loss of ARID1A in some cancers (28). ARID1A and ARID1B are frequently co-mutated in cancer, but ARID1A-deficient cancers retain at least one functional ARID1B allele. The result suggests that loss of ARID1A and ARID1B alleles (double knockout) together promotes aggressive carcinogenesis, followed by dedifferentiation and hyper-proliferation in the liver and skin (29). Since 10.67% of melanoma patients have altered ARID1A, this may be useful in identifying ARID1B as a potential therapeutic target in patients with ARID1A loss or alteration.

SMARCA4 (BRG1) and SMARCA2 (BRM) are the two critical components of SWI/SNF ATPases that use ATP to generate energy for nucleosome remodeling, which is often mutated or silenced in cancer (30). The first somatic genetic alteration of *SMARC2* was found in human non-melanoma cancer. Both act as tumor suppressors by controlling the cell cycle and regulating adhesion between cells. Several studies have also reported a synthetic lethality relationship between these two (19, 31). A 2018 study by Reyes and Martinez reported that high expression of SMARCA4 correlated with aggressive tumors, while high expression of SMARCA2 was associated with benign differentiated tumors. They showed that the expression of these two factors has a high prognostic value and high expression of SMARCA4 was significantly associated with poor prognosis and survival in uveal melanoma (32, 33), with high expression occurring in later stages of metastatic melanoma (34). Furthermore, one of these subunits is required for melanoma tumorigenesis (33, 35).

The master regulator of melanocyte differentiation from progenitor cells and survival, *microphthalmia-associated transcription factor (MITF)*, showed several interactions with the SWI/SNF complex. The *MITF* gene plays a cooperative role with the subunits of the complex to promote tumorigenesis, and on the other hand, there is evidence that some SWI/SNF subunits are downregulated (4). SMARCA4 regulates MITF, melanin synthesis, protection against UVR damage, and survival in melanocytes and melanoma cells (36). SMARCA2 does compensate for the function of SMARCA4 in the case of a loss-of-function mutation associated with MITF (37), but SMARCA2 only partially compensates for the loss of SMARCA4 when all MITF targets are expressed (38). In addition to SMARCs, a recent study by Trivedi et al. (39) has shown that inhibition of bromodomain and extra-terminal (BET) by JQ1 can lead to decreased expression of MITF targets.

### 3.2. Druggable pathway targets

In a recent review article by Guo et al. (40) describing the signaling pathways involved in melanoma, including the mitogen-activated protein kinase (MAPK) pathway, protein kinase B (AKT) pathway, cell cycle regulation pathway, pigmentation-related pathway, and p53 pathway, epigenetic factors were also mentioned as one of the crucial factors in melanoma carcinogenesis. In melanoma, mutations in BRAF (50%−70%), NRAS (15%−30%), NF1, KRAS and HRAS (in 2 and 1% of patients, respectively), and KIT are responsible for MAPK dysregulation. Activation of the MAPK pathway leads to cell proliferation, invasion, metastasis, survival, and angiogenesis (41). BRAF (serine/threonine kinase), which is a member of the Raf family, plays a critical role in MAPK pathway signaling, with a substitution from valine to glutamic acid at codon 600 (V600E) in ~50% of melanomas (40). The FDA approved vemurafenib (PLX4032) and dabrafenib for the treatment of metastatic melanoma in 2011 and 2013, respectively. The drugs specifically target patients whose tumors have the BRAF V600E gene mutation (Table 1). The phosphatidylinositol 3-kinase (PI3K)/protein kinase B (Akt) pathway, which has more or less the same functions in cells, is another metabolic pathway that is dysregulated in melanoma, although there is no FDA-approved agent that directly inhibits this pathway in melanoma, however, there are several studies showing that synthetic small molecule compounds have an effect on PI3K/AKt and/or PI3K/Akt/mTOR and the associated RAS/RAF/MEK/ERK or MAPK pathway, such as NVP-BEZ235 (53) and Rapalogs (Everolimus, Deforolimus, and Temsirolimus) (54). c-KIT or CD117 is a transmembrane receptor tyrosine kinase (RTK) identified in hematopoietic cells, germ cells, gastrointestinal tract Cajal cells (GI), melanoma cells, B-cell progenitor cells, and mast cells that sends signals to maintain survival, promote cell proliferation, differentiation and regulate growth and development (55). Approximately 70% of KIT mutations in melanoma are missense mutations (well described in reference (55)). Nilotinib, Dasatinib, Sunitinib, and Masitinib are the small molecules that target cKIT and are currently being tested in clinical trials. It should be noted that pathway inhibitors have shown a potential synergistic effect with other antitumor agents in melanoma such as checkpoint inhibitors (56) and anti-cytotoxic T-lymphocyte antigen 4 (CTLA-4) antibodies (57).

**Table 1:** Druggable targets in melanoma-the FDA, clinical trial, or assessment status of different compounds in melanoma.

**Mechanism of action**	**Compound**	**Status in melanoma**	**References**
Anti–PD-L1	Nivolumab	On 18 March, 2022, the FDA approved nivolumab and relatlimab-rmbw (Opdualag, Bristol-Myers Squibb Company) for adult and pediatric patients 12 years of age or older with unresectable or metastatic melanoma	Sahni et al. (42)
	Pembrolizumab	FDA approves Merck's KEYTRUDA^®^ (pembrolizumab) as adjuvant treatment for adult and pediatric (≥12 years of age) patients with stage IIB or IIC melanoma following complete resection	–
HDAC inhibitor	Domatinostat (*4SC-202*)	FDA approves IND application for Domatinostat (*4SC-202*) in melanoma	–
	Entinostat	Phase II	An exploratory study of pembrolizumab plus entinostat in non-inflamed stage III/IV melanoma: https://clinicaltrials.gov/ct2/show/NCT03765229
	Azacytidine	Phase II	Study of oral azacitidine (CC-486) in combination with pembrolizumab (MK-3475) in patients with metastatic melanoma: https://clinicaltrials.gov/ct2/show/NCT02816021
	Tinostamustine	Phase I	Tinostamustine and nivolumab in advanced melanoma (ENIgMA) https://www.clinicaltrials.gov/ct2/show/NCT03903458
	ACY-241	Phase I	Selective HDAC6 inhibitor ACY-241 in combination with ipilimumab and nivolumab https://clinicaltrials.gov/ct2/show/NCT02935790
EZH2 inhibitors	Tazemetostat	The FDA has approved Tazverik (tazemetostat) on 24 January, 2020, is marketed by Epizyme Inc. to treat adults and children 16 and older with epithelioid sarcoma, Tazverik is only the second targeted therapy (https://www.cancer.org/cancer/soft-tissue-sarcoma/treating/targeted-therapy.html) approved for soft tissue sarcoma and the first treatment option specifically for epithelioid sarcoma (https://www.cancer.org/cancer/soft-tissue-sarcoma/about/soft-tissue-sarcoma.html)	—
	GSK503	*In vitro*	(https://www.ncbi.nlm.nih.gov/pmc/articles/PMC6174981/pdf/PATH-245-433.pdf) and https://pubmed.ncbi.nlm.nih.gov/25609585/
Anti-cytotoxic T lymphocyte antigen 4 (CTLA-4) antibodies	Ipilimumab	FDA approves YERVOY™ (ipilimumab) for the treatment of patients with newly diagnosed or previously-treated unresectable or metastatic melanoma on March 25, 2011	—
	Tremelimumab	Phase III	Ribas et al. (43)
BET (bromodomain and extra-terminal) inhibitors	JQ1	*In vitro*	Trivedi et al. (39)
	NHWD-870	*In vitro*	Deng et al. (44)
RVX2135 or iBET762	*In vitro*	Muralidharan et al. (45)
BRAF inhibitors (serine/threonine kinase)	Sorafenib	Phase II	Eisen et al. (46)
	Vemurafenib (PLX4032)	FDA approves vemurafenib (PLX4032) on 18 Aug 2011, for treatment of metastatic or unresectable melanoma. The drug specifically targets patients whose tumors express the BRAF V600E gene mutation	–
	Dabrafenib or GSK2118436	The FDA approved dabrafenib as a single-agent treatment for patients with BRAF V600E mutation-positive advanced melanoma on May 30, 2013	Ballantyne et al. (47)
	RAF-265 (formerly CHIR-265)	Phase I	Harris (48)
	XL281	Phase I	https://clinicaltrials.gov/ct2/show/NCT00451880
C-kit tyrosine kinase activity inhibitors	Imatinib	Phase III	Wei et al. (49)
	Sunitinib	Phase II	https://www.clinicaltrials.gov/ct2/show/NCT00631618
	Dasatinib	Phase II	https://clinicaltrials.gov/ct2/show/NCT00700882 https://clinicaltrials.gov/ct2/show/NCT00436605
	Nilotinib	Phase I	https://clinicaltrials.gov/ct2/show/NCT04903119
Histone deacetylase inhibitors	SAHA	*In vitro*	Basu et al. (50)
Bromodomain inhibitor	PFI-3 (selective SMARCA2/4 bromodomain inhibitor )	*In vitro*	Yang et al. (51)
	TP-472 (Inhibition of BRD9)	*In vitro*	Mason et al. (52)

### 3.3. Drugs currently under clinical trials

The results of Martí et al. from 2012 show that the potential target therapies in melanoma can be divided into two categories: first, the strategy may target the tumor cell using molecules that can inhibit growth and/or prevent cell death, or molecules responsible for facilitating invasion and/or metastasis. The second category targets structure rather than cells, such as angiogenesis and immune tolerance. They reported BRAF inhibitors (Sorafenib, PLX4032, GSK2118436, RAF-265, XL281), inhibitors of c-kit tyrosine kinase activity (Imatinib, Sunitinib, Dasatinib, Nilotinib), and anti-cytotoxic T-lymphocyte antigen 4 (CTLA-4) antibodies (Ipilimumab and Tremelimumab), which showed the best test results in patients with melanoma (57). Histone deacetylase inhibitors (HDACI) are the enzyme with anticancer activity, the only agents approved in the clinic for melanoma^2^, which inhibit histone deacetylases (HDAC) to inhibit tumor cell proliferation (58). Domatinostat (59), Entinostat^3^, Azacytidine^4^, ACY-241, and Tinostamustine^5^ can be placed in this category, which has been repeatedly demonstrated in preclinical and clinical studies. Garmpis et al. (58) proposed to investigate the synergistic effect of HDACi (Vorinostat (5AIIA, Zalmza), Romidepsin (Istodox, Depsipeptide), Belinostat and Panobinostat (Farydak) with other inhibitors such as BRAF inhibitors and BET inhibitors, which may lead to melanoma treatment. According to this study, the combination of the HDAC inhibitor LBH589 and the BET inhibitor I-BET151 showed apoptosis of melanoma cells but not of melanocytes *via* the mitochondrial pathway in the AKT and Hippo/YAP signaling pathways. This study demonstrated the effect of combination therapy on melanomas, including those resistant to BRAF inhibitors (60). *In vitro* treatment with the histone deacetylase inhibitor Vorinostat (SAHA) resulted in suppression of MITF and cell death in melanocytic nevi (50). A clinical trial of PDR001 and the HDAC inhibitor Panobinostat in patients with metastatic melanoma who have failed prior anti-PD1 or PD-L1 therapy was registered in 2019. The trial was withdrawn by the decision of the sponsor, but another trial evaluating the combination of the HDACi fusion molecule Tinostamustine (EDO-S101) and the anti-PD-1 monoclonal antibody Nivolumab in patients with refractory, locally advanced or metastatic melanoma was initiated in 2019 and is enrolling patients.^6^ Since 2010, the FDA has approved a number of therapeutic agents and synergistic approaches against melanoma, including Ipilimumab, Nivolumab, Pembrolizumab and the combination of Ipilimumab and Nivolumab, Vemurafenib, Dabrafenib, the combination of Dabrafenib plus Trametinib, Vemurafenib plus Cobimetinib, and Encorafenib plus Binimetinib (well described in reference (40)). Table 1 shows the information on agents tested or approved for melanoma, categorized by the mechanism of action, most of which have been discussed in this article.

The new strategy of combination therapy, especially in combination with BET inhibitors, showed interesting results in overcoming patient relapse and resistance after treatment with approved drugs targeting the MAPK pathway. For example, a recent study (44) showed that secreted phosphoprotein 1 (SPP1) expression can be a melanoma driver, and BET inhibitor NHWD-870 targets the BRD4 subunit and suppresses SPP1 expression and ultimately melanoma progression *via* the non-canonical NF-κB/SPP1 pathway (44). BET inhibitors (RVX2135 or iBET762) in combination with ATRIs [Ataxia-telangiectasia and Rad3-related (ATR) is a kinase belonging to the PI3 kinase-like family; VE821 or AZ20], showed apoptosis effect on melanoma cells as well as the PDX model of melanoma (45). Another study by Paoluzzi et al. (61) showed that the BET inhibitor JQ1 in combination with the BRAF inhibitorV suppressed tumor growth and significantly improved survival compared to either drug alone. Vemurafenib demonstrated safety and efficacy in both treatment-free and pre-treated BRAF-mutated melanoma patients (62).

In this section, we have attempted to provide an update on agents tested or approved for melanoma, most of which target the SWI/SNF complex. However, it should be noted that some *in vitro* or preclinical studies have shown the novel potential of new small molecules, e.g., the study by Zingg et al. (63), showed that EZH2 levels are upregulated in cancer cells after anti-CTLA-4 or IL-2 immunotherapies, leading to loss of tumor control. In this study, combination therapy with EZH2i (i.e., GSK503) may restore tumor immunogenicity. In general, and after reviewing various evidence that showed a contrast in the use of EZH2 inhibitors in cancers, especially mesothelioma, it seems that testing the response to EZH2 should be carefully evaluated before using these molecules as therapy (64). Recent research has shown that a novel bromodomain inhibitor called PFI-3, which targets SWI/SNF, and is responsible for repairing double-strand breaks in cancer, synergistically sensitizes many human cancers cell lines against DNA damage caused by chemotherapeutic agents such as doxorubicin (65). PFI-3 is a selective, potent, and cell-permeable SMARCA2/4 bromodomain inhibitor that has been previously characterized in the setting of various cancers (e.g., lung cancer, synovial sarcoma, leukemia, and rhabdoid tumors) (66). SMARCA2/SMARCA4 ATPases simulate synthetic lethality through multiple inhibitors for cancers containing a BRG1 loss-of-function mutation, as described in the 2018 study by Papillon et al. (67). It should be noted that SMARCA4, SMARCA2, BRD7, BRD9, and PBRM1 contain drug-acting bromodomains that showed interaction with synthetic lethality to serve as therapy. TP-472, a compound with selective function and similarity to the bromodomain of BRD7 and BRD9, has antitumor activity on melanoma (68).

## 4. SWI/SNF complex as a targeted therapy in other cancers

Mutations in SWI/SNF's subunits are reported in ~25% of cancers (69). Although this paper is focused on melanoma, this section tries to show the footprint of this complex's mutation in different cancer. A comprehensive review by Centore et al., was published in 2020 based on the large-scale cancer genome-sequencing studies showed targeted therapies in different cancers based on this complex mutation, for example, ARID1A mutation as a hallmark in the bladder, stomach, and endometrial cancers which targeted by ARID1B selective degrader, EZH2 inhibitors and P13K inhibitors. SMARCA4, in nonsmall cell lung carcinoma, was targeted through the synthetic lethal pathway and by targeting SMARCA2 inhibitors (70). In Silico analysis of the SWI/SNF complex shows 70% of mutations with functional impact on lung adenocarcinoma patients (71). SMARCA2 in esophageal, SMARCB1 in malignant rhabdoid tumor and epithelial sarcoma, and PBRM1 in kidney cancer are collectively mutated and reported. These mutations are targeted by SMARCA4-selective inhibitors, BRD9-selective degraders, EZH2 inhibitors, and immune checkpoint inhibitors, respectively (70). On other hand, some of the studies focused on the ATPase part to conduct target therapy, degradation of ATPase subunit of SWI/SNF can disrupt physical chromatin accessibility to disable oncogenic transcription (for instance in prostate cancer) (72), and BRM as a core ATPase subunit is downregulated in hepatocellular carcinoma (HCC), colorectal and gastric cancer, small cell carcinoma of the ovary (SCCOHT), ovarian clear cell carcinoma (OCCC), non-small cell lung cancer (NSCLC), adenocarcinoma of the lung (AD), large cell carcinoma of the lung (LC), pleomorphic carcinoma of the lung (PL), clear cell renal cell carcinoma (ccRCC), and non-melanoma skin cancer (NMSC) (73). It should be noted that BRM as well as some of the therapeutic agents to target these complex acts context-dependent (as we also mentioned above about liver cancer) (74). Considering context dependency, cancer dependent-specific study is required to validate therapeutic agents targeting SWI/SNF complex subunits.

## 5. Conclusion and future direction

A theme developed from recent studies showed the crucial role of the SWI/SNF complex in defining the therapeutic efficacy of melanoma. In light of accumulated data, *ARID2, ARID1B, SMARCA4 (BRG1)*, and *SMARCA2 (BRM)* have the most important mutations in melanoma. Considering the important role of epigenetic players in immune therapy resistance in a patient with melanoma. It is crucial to determine how SWI/SNF complex can contribute to melanoma therapy through different subunits. Combinational therapy and synthetic lethality approaches are the well-studied most current findings that show promising clinical responses in melanoma. Of note, further investigations need to be done to elucidate the context-dependent behavior of SWI/SNF subunits, possible off-target inhibition, immunosuppression, and the chance of relapse in target therapy for melanoma. Targeting the druggable SWI/SNF bromodomains (BRD7, BRD9, SMARCA4, SMARCA2), using the BET inhibitors as long as the HDAC inhibitors and identification of synthetic lethal interactions involved in melanoma such as SMARCA4 and ARID2 presents an additional possibility for novel strategies targeting the SWI/SNF subunits toward precise medicine of melanoma. Given the significant role of the SWI/SNF complex in melanoma, future therapeutic approaches must focus on mechanisms of synergic effect and synthetic lethality to enhance the therapeutic benefits of inhibitors, particularly when there is a deficiency in the functional domains mentioned above. We strongly believe an understanding of potential therapeutic vulnerabilities based on SWI/SNF in melanoma is leading to personalized and targeted cures and opening up new areas of clinical investigations.

## Author contributions

Conception and design of study: MM and MA. Acquisition of data and revising the manuscript critically for important intellectual content: MA, MN, and MM. Drafting the manuscript: MM. All authors contributed to the article and approved the submitted version.

## Footnotes

^1^ https://clinicaltrials.gov/ct2/show/NCT02601950

^2^ https://clinicaltrials.gov/ct2/show/NCT03022565

^3^ https://clinicaltrials.gov/ct2/show/NCT03765229

^4^ https://clinicaltrials.gov/ct2/show/NCT02816021

^5^ https://clinicaltrials.gov/ct2/show/NCT03903458

^6^ https://www.clinicaltrials.gov/ct2/show/NCT03903458
